# Fungal Based Biopolymer Composites for Construction Materials

**DOI:** 10.3390/ma14112906

**Published:** 2021-05-28

**Authors:** Iuliana Răut, Mariana Călin, Zina Vuluga, Florin Oancea, Jenica Paceagiu, Nicoleta Radu, Mihaela Doni, Elvira Alexandrescu, Violeta Purcar, Ana-Maria Gurban, Ionela Petre, Luiza Jecu

**Affiliations:** 1 National Institute for Research & Development in Chemistry and Petrochemistry—ICECHIM, 202 Independentei Spl., 060021 Bucharest, Romania; iulia_rt@yahoo.com (I.R.); marriconstantin@yahoo.com (M.C.); florino@ping.ro (F.O.); nicolbiotec@yahoo.com (N.R.); mihaela.doni@icechim.ro (M.D.); elviraalexandrescu@yahoo.com (E.A.); purcar.violeta@icechim.ro (V.P.); amgurban@icechim.ro (A.-M.G.); 2 Doctoral School of Biotechnology, University of Agronomic Sciences and Veterinary Medicine of Bucharest, 59 Marasti Bvd, District 1, 011464 Bucharest, Romania; 3 CEPROCIM S.A., 6 Preciziei Street, 062203 Bucharest, Romania; jpaceagiu@yahoo.com (J.P.); ionela.petre@ceprocim.ro (I.P.)

**Keywords:** biopolymer composites, *Ganoderma lucidum*, *Bacillus amyloliquefaciens*, fungal mycelium

## Abstract

Environmental contamination, extensive exploitation of fuel sources and accessibility of natural renewable resources represent the *premises* for the development of composite biomaterials. These materials have controlled properties, being obtained through processes operated in mild conditions with low costs, and contributing to the valorization of byproducts from agriculture and industry fields. A novel board composite including lignocelullosic substrate as wheat straws, fungal mycelium and polypropylene embedded with bacterial spores was developed and investigated in the present study. The bacterial spores embedded in polymer were found to be viable even after heat exposure, helping to increase the compatibility of polymer with hydrophilic microorganisms. Fungal based biopolymer composite was obtained after cultivation of *Ganoderma lucidum* macromycetes on a mixture including wheat straws and polypropylene embedded with spores from *Bacillus amyloliquefaciens*. Scanning electron microscopy (SEM) and light microscopy images showed the fungal mycelium covering the substrates with a dense network of filaments. The resulted biomaterial is safe, inert, renewable, natural, biodegradable and it can be molded in the desired shape. The fungal biocomposite presented similar compressive strength and improved thermal insulation capacity compared to polystyrene with high potential to be used as thermal insulation material for applications in construction sector.

## 1. Introduction

Traditional polymeric materials used for common applications as insulating and packing are replaced by new generation of materials based on biological materials. Microorganisms have extraordinary properties, namely versatility and rapid growth on cheap substrates at mild conditions that recommend them as suitable materials for these types of applications. Thereby, fungal mycelium that is an inert, nontoxic and safe material has become a viable alternative to obtain an eco-friendly biocomposite material. Mycelia of filamentous fungi provide mechanical strength to panels and boards, fire resistance, acoustical and thermal insulating properties. The use of agro byproducts as fungal cultivation substrate, as well as filler for biomaterial has the benefit of reducing the negative impact on the environment regarding the disposable of agro-industrial wastes.

For construction applications, spongy structure formed by growing fungi on the support of bio-agro-byproducts must have several properties, such as mechanical strength, thermal insulation capacity and a lower biodegradation rate. The thermal conductivity of the biopolymer-based thermal insulation material must not exceed the value between 0.03 and 0.045 W/mK, in order to ensure a competitive character in relation to the other insulating materials (e.g., glass wool 0.04 W/mK, wool mineral 0.04 W/mK, expanded polystyrene 0.038 W/mK, extruded polystyrene 0.035 W/mK, etc.).

The thermal conductivity of biomaterial depends on the thermal conductivity of the components. For this purpose, plastics and especially polyolefins can be used. Among polyolefins, polypropylene (PP) is a polymer with balanced physical and mechanical properties, very good electrical insulator, being easy to process and possessing a low thermal conductivity. Common plastics, especially those based on polyolefins, have hydrophobic surfaces, which have low compatibility with hydrophilic microorganisms. The increase of their biocompatibility, which ensures a subsequent slow biodegradation, was achieved by increasing the hydrophilicity of the surfaces, either by photooxidation [1], by gamma irradiation [2], or by bacteria incorporation [3]. Bacteria embedded in the plastic migrates to the outside and form hydrophilic biofilms that stimulates the growth of fungal mycelium.

Another interesting solution to improve the compatibility between nanocelullose and PP for sustainable polypropylene nanocomposites was achieved by modifying supermolecular structure and dispersing the morphological properties of the nanocellulose component under controlled enzymatic hydrolysis using cellulases from powerful manufacturers of cellulolytic system, and microscopic fungi such as *Trichoderma reesei* and *Aspergillus* sp. [4].

Among the fungi, *Pleurotus ostreatus*, *Ganoderma lucidum*, *Lentinula edodes*, and *Coprinus comatus* represent fungal species active in degradation of substrates containing lignocelluloses complexes, especially hemicelluloses and lignin, based on secreted enzymatic complex systems [5,6,7]. Thus, filamentous *basidiomycetes Ganoderma lucidum*, a common environmental fungus was selected to design a thermal insulating biomaterial. *Ganoderma* is one of the oldest mushrooms with pharmacological activity and medicinal uses, *Ganoderma lucidum* being able to produce a biomass rich in bioactive compounds [8,9,10].

In addition to medicinal uses, since the fungus grows as a dense white mycelium in desirable shape, it is possible to be used as substrate support, intended for filling panels and boards. The mycelium produced by *Ganoderma* is a dense hyphae network, with a solid three-dimensional arrangement open to the passage of gases and liquids. The architecture, composition of cell wall, constituents and rates of growth are depending on several factors. Due to these features, fungal foam from *Basidiomycetes* is considered an alternative to synthetic foam expanded polystyrene, possessing the advantage of being biodegradable [11,12]. However, high biodegradability rate is not desirable for practical applications, because it could be associated with hazards, such as development of the deleterious fungi and/or insect [13]. The use of the recycled plastic network based on polyolefins could greatly improve both, mechanical and decays resistance properties. A drawback of this approach is the low compatibility between hydrophobic polyolefins and mycelium based hydrophilic material.

In this respect, the present work aimed: (1) to obtain mycelium composites from a fungal strain grown on mixture of agro-industrial byproducts and synthetic polymer compatibilized by embedding bacterial spores, and (2) to characterize morphologically, thermally and chemically the mycelium composites, in order to assess the material properties and to evaluate the potential for its use in thermal insulation in construction field.

## 2. Materials and Methods

### 2.1. Microorganisms

There were used the following strains: *Bacillus amyloliquefaciens* 1014 from Microbial Collection of ICECHIM, *Ganoderma lucidum* purchased from German Collection of Microorganisms and Cell Cultures (DSMZ) (Braunschweig, Germany).

### 2.2. Conditions of Microbial Cultivation

**Figure 1:**
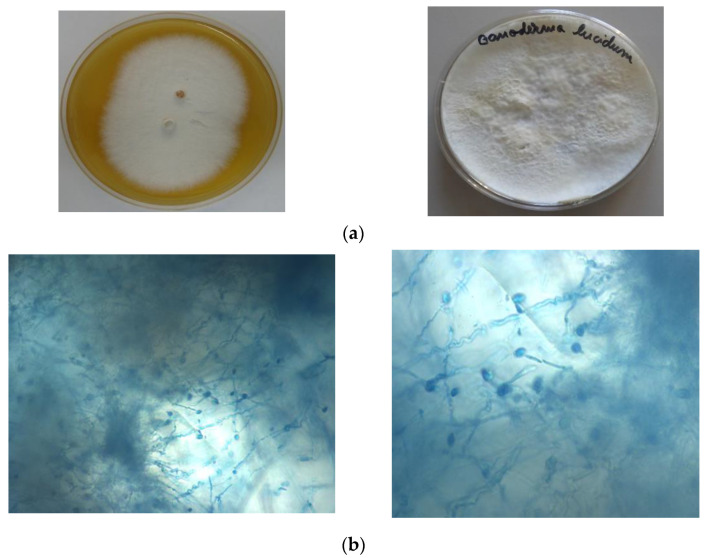
Macro- and microscopic observations of *Ganoderma lucidum* culture. (**a**) Macroscopic observations of *Ganoderma* cultures on malt-peptone agar medium. Mycelium fungus grown in the laboratory; (**b**) Microscopic observations (Olympus BX 51); spores and fungal filaments, lactophenol staining (×20 left, ×40 right).

*Bacillus amyloliquefaciens 1014* was cultured on Luria Bertani (LB) medium with the following composition (g L^−1^): 10, tryptone; 5, yeast extract; 10, NaCl; 15, agar [14]. The preinoculum was obtained by cultivation on LB medium without agar. The nutrient medium for *Bacillus* sporulation had the composition (g L^−1^) [15]: l12.7, lactose; 16.7, starch; 1.8, (NH_4_)_2_SO_4_; peptone; mineral solution (2.0, KH_2_PO_4_; 1.0; K_2_HPO_4_; 0.2, MgSO_4_·7H_2_O; 0.01, CaCl_2_·2H_2_O; 0.045, FeSO_4_·7H_2_O; 0.001, ZnSO_4_·7H_2_O; 0.001, MnSO_4_·4H_2_O); pH medium = 7–8. *Bacillus* was cultured in 300 mL Erlenmayer flasks with 150 mL medium inoculated with 2% sporal suspension, and cultured on incubator Heidolph Unimax 101, at 34 °C, 200 rpm, for 48 h (Figure 1). Broth culture was centrifuged at 4000 rpm and the pellet was processed at atomizer. *Ganoderma lucidum* was cultured in Petri plates on medium with the following composition (g L^−1^): 30, malt extract; 3, peptone; 15, agar. The plates were incubated at 30 °C for 5 days. Microscopically observations were made at microscope Olympus BX 51.

### 2.3. Spray-Drying of Bacterial Spores

The bacterial pellets were resuspended in phosphate buffer 0.1 M, (KH_2_PO_4_/K_2_HPO_4_,) pH = 7, mixed with maltodextrin in various proportions (Table 1) and spary-dryed by using a laboratory spray-dryer (Buchi B-290, Buchi, Flavil, Switzerland), at 140 °C entrance temperature and 80 °C exit temperature (Table 1).

### 2.4. Preparation of Composites Consisting in Atomized Bacterial Pellet and Polypropylene (PP)

PP homopolymer Moplen HP500N produced by LyondellBasell Polymers with a melt flow index of 12 g/10 min (230 °C/2.16 kg) and a density of 0.90 g/cm^3^ was used as matrix for composite preparation.

Polypropylene thin films (thicknesses less than 0.1 mm) with embedded bacterial spores were prepared. The films were obtained by hot pressing using a laboratory press, at 190 °C and 200 bar, resulting a “sandwich” consisting of two PP films between which the bacterial spores were evenly distributed in a proportion of 6% of the final product.

### 2.5. Viability of Bacterial Spores

The viability of bacterial spores was verified using a suspension of atomized preparate in sterile water which was inoculated in Petri plates on LB medium, for 24 h, at 32 °C. The bacterial growing cultures were examined using an Olympus BX51 microscope. Also, the viability of spores included in polypropylene and processed at temperatures of 190 °C was verified. The polymeric samples were sterilized by immersion in ethanol, rinsed with sterile water and incubated on liquid LB medium at 24 °C.

### 2.6. Antagonism between Ganoderma lucidum and Bacillus amyloliquefaciens 1014

The test was carried out according to classical dual method [16]. The microorganisms were inoculated juxtaposed in Petri plates on agar medium (g L^−1^) (30, malt extract; 3, peptone; 15, agar). Since *Ganoderma* is characterized by a slower growth comparative with *Bacillus* *amyloliquefaciens 1014*, the fungus was inoculated as a 5 mm mycelium fragment 48 h before bacteria. Antagonistic activity was evaluated after 3–5 days of cultivation using the following formula:

X = I_A_/I_B_ × E_B_/E_A_, (1)

where, I = internal radius; E = external radius; A = microorganism test (*Ganoderma lucidum*); B = microorganism antagonist (*Bacillus amyloliquefaciens 1014).* The obtained results are considered as antagonism present for X < 1, and antagonism absent for X > 1.

### 2.7. Preparation of Biomaterials Boards

Wheat straws (90% w/w), strips of polypropylene embedded with bacterial spores (10% w/w) were mixed in plastic autoclavable bag, moistened with 200 mL of nutrient solution (per each board) and inoculated with mycelial fragments of *Ganoderma* cultures on nutrient agar medium. Nutrient solution had the following composition (g L ^−1^): 30, extract de malt (Merck); 3.0, peptone (Scharlau). Polypropylene strips (0.5 cm × 0.5 cm) were sterilized with 70% ethanol, rinsed with sterile water and then exposed to UV radiation for 10 min. The final size of material board was 30 × 30 × 1.5 cm (height). The mixture was placed in rectangular wooden shape and incubated at 30 °C for 30–35 days. After that period, the boards were sterilized in autoclave to kill any hyphae or biological material in composite and dried in a convection oven at a temperature of 80 °C, for 5 to 10 h, until their weight stabilized. The decrease of board weight after drying was about 5–7% (w/w).

### 2.8. FTIR Analysis

FTIR measurements of the samples were performed using a Spectrometer Tensor 37 (Bruker Instrument, Woodstock, NY, USA), in Attenuated Total Reflectance mode (ATR, Golden Gate diamond unite). All samples were recorded in the wavelength range of 4000–500 cm^−1^, averages of 64 scans per spectrum, with the resolution of 4 cm^−1^.

### 2.9. Thermal Conductivity

The thermal conductivity (λ, W/mK) was calculated based on the thermal resistance (Rt, m^2^ K/W) of composites, using a hot plate apparatus type EP500 manufactured by Lambda–Messtechnik, in the temperature range 10–40 °C. The temperature difference (Δt) between the hot plate and the cold plate of the apparatus was 15 degrees K.

Rt = δ/λ
(2)

where δ is sample thickness (mm)

(3) $$\lambda =\dot{q}\cdot \frac{\delta \cdot 10^{-3}}{\Delta t}\;\left[W/\mathrm{mK}\right]$$

where $\dot{q}$ is unit heat flow (W/m^2^).

The measurements on plates were done in triplicate.

### 2.10. Mechanical Testing in Compression

The compressive strength (kPa), at 25% deformation, was determined according to ISO 844, at 25 °C and 50% relative humidity, with 1 mm/min, using an Instron 3382 Universal Testing Machine and three rectangular specimens of 50 × 50 × 30 mm for each sample.

## 3. Results

### 3.1. Microbial Cultivation

The characteristics of *Ganoderma* cultures are observed, namely, slightly fluffy to powdery obverse (initially white, later turning white-yellow), with beige reverse (Figure 1a).

### 3.2. Viability of Bacterial Spores Embedded in Polypropylene Matrix

**Figure 2:**
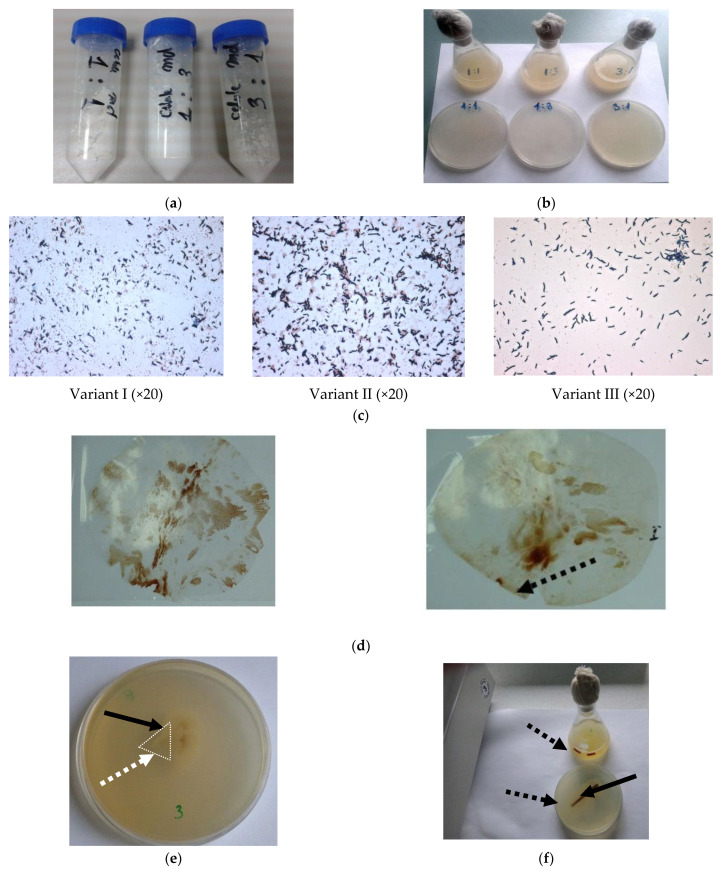
Testing the viability of *Bacillus* spores in various mixtures, (**a**,**b**) atomized preparate; (**c**,**d**) composite pieces. (**a**) Atomized bacterial—dextrin mixtures; (**b**) Solid and liquid cultures on LB medium inoculated with atomized preparate; (**c**) Microscopically observations of viable spores from atomized bacterial—dextrin mixtures; (**d**) *Bacillus amyloliquefaciens* 1014 spores embedded in polypropylene. (dotted arrow—polypropylene piece sampling); (**e**) Cultivation on solid LB medium (white square and dotted arrow—polypropylene sample from Figure 2d placed on nutrient medium; black arrow—bacterial growth from viable spores); (**f**) Cultivation on solid and liquid LB medium (dotted arrow—polypropylene sample placed on/in nutrient medium; black arrow—bacterial growth from viable spores).

The viability of bacterial spores was investigated by plating polymer embedded with bacterial spores on the rich medium. There were studied spores included in atomized preparate (Figure 2a), as well as those embedded in polypropylene pieces (Figure 2d,e).

Regarding the different ratio of maltodextrin and bacterial pellet, the optimal composition was variant II (10 g maltodextrin and 10 g bacterial pellet in 60 mL phosphate buffer) which has been selected for further experiments since the microscopically observations revealed a higher density of bacterial spores in examined atomized samples (Figure 2b,c). As a general remark, it is important to underline that *Bacillus* spores were found to be viable even after hot pressing the sandwich containing the bacterium spores between two thin PP films.

### 3.3. Antagonism Fungus-Bacteria

**Figure 3:**
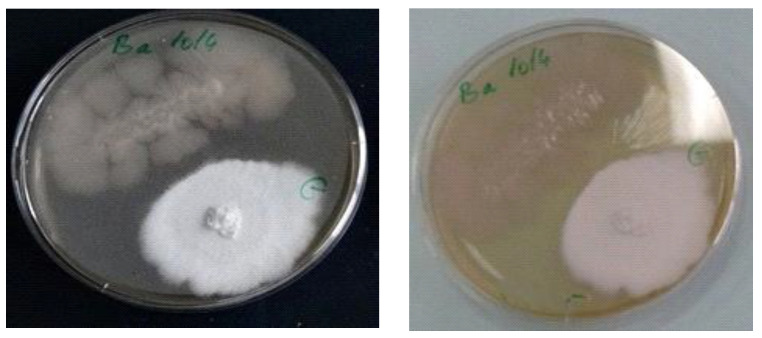
Images of experimental study regarding the antagonism between *Bacillus Amyloliquefaciens 1014* and *Ganoderma lucidum*.

The antagonism test was carried out by juxtaposed cultivation of microorganisms in Petri plates on agar medium. As it cand be seen in Figure 3, no antagonism between fungus and bacteria was found.

Each microorganism has developed maintaining the growth zone, without invasions, overlaps or growth inhibitions. No mechanism of induced defense, as secondary metabolites like antibiotics or mycotoxins, and lysis of microbial cells were observed. This is an encouraging result for the preparation of insulating biomaterial, which involves the inclusion of *Bacillus* spores in the polypropylene matrix and *Ganoderma* cultivation on mixed substrate, including wheat straws and polymer composites.

### 3.4. Preparation of Biomaterials Boards

**Figure 4:**
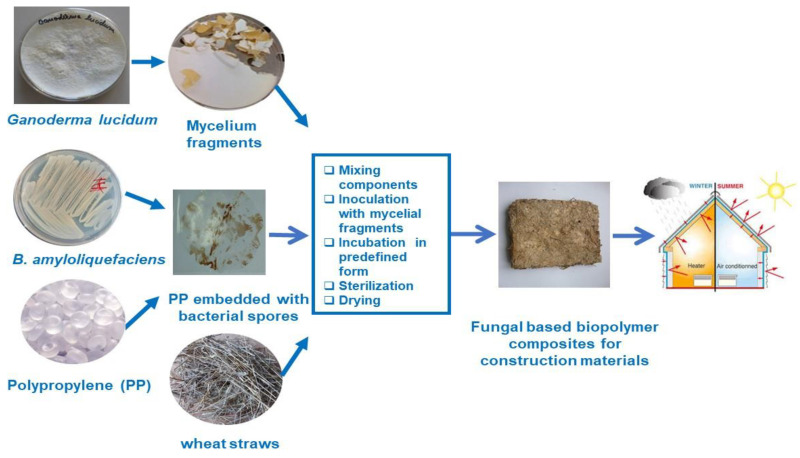
Schematic representation for preparation of fungal composite material.

The stages of biomaterial obtainment are described in Figure 4.

**Figure 5:**
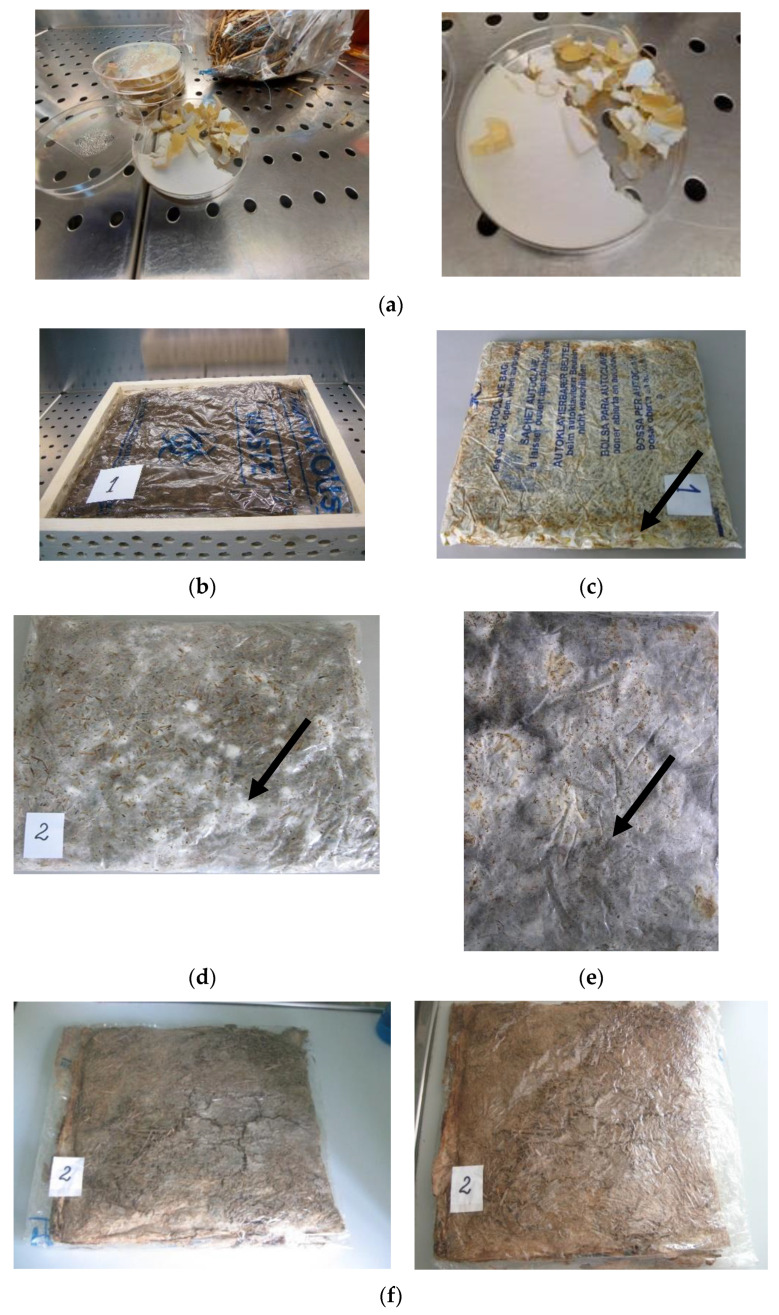

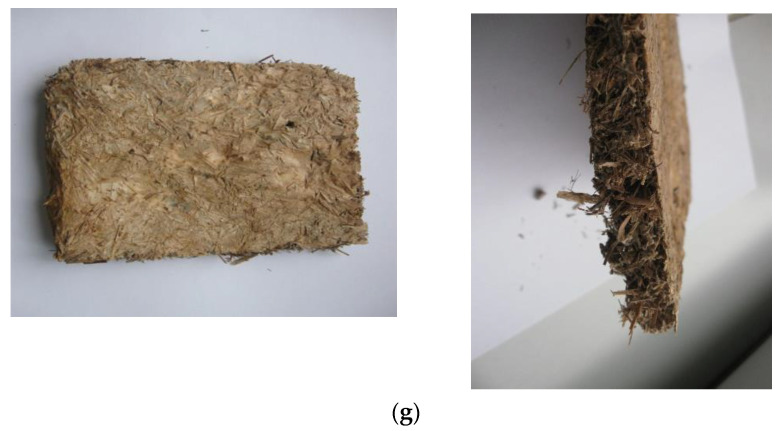
Preparation of biomaterial board based on fungal mycelium grown on lignocellulosic substrat (wheat straws) and polypropylene embedded with bacterial spores. (**a**) Fragments of *Ganoderma* mycelium prepared for boards inoculation; (**b**) Cultivation in rectangular predefined form; (**c**) Cultural bag with mycelium growth in progress (one week of incubation; arrow—white fungal mycelium); (**d**) Cultural bag with mycelium growth in progress (three weeks of incubation; arrow—white fungal mycelium); (**e**) Cultural bag with mycelium growth in progress (four weeks of incubation; arrow—white fungal mycelium); (**f**) Biomaterial board after autoclave sterilization; (**g**) Final biomaterial board (after sterilization and drying at 80 °C). Mycelium composite samples.

For the next stages of biomaterial preparation, the nutrient medium with *Ganoderma* mycelium was cut into small pieces and used to inoculate the substrates (Figure 5a). As it can be expected, the growth of *Ganoderma* started from the sowing mycelium pieces (Figure 5d,e), developing a white mycelium with hyphae penetrating between the wheat straw and the polymer strips. After mycelium colonization, the wheat straws and PP pieces were covered by a fibrous network of hyphae. Also, the color of whole mixture become brown, phenomenon attributed to the release of melanin, the production of this pigment being a characteristic for the decay of lignocellulosic substrates by basidiomycetes [17,18].

The fungal growth was quite slow because it occurred in a relatively closed space with limited aeration at the pores of the polypropylene bag. To a certain extent, *Ganoderma* growth was stimulated by wetting the mixture from autoclavable bag with the nutrient solution. An important contribution to fungal growth was achieved by the presence of bacteria forming biofilm which sustain the fungal growth without any antagonism, and improving the compatibility between polymer and fungal hyphae.

The growth process lasted 20–25 days and this long colonization leaded to stiffer and stronger structure. The dense hyphae networks that reinforced the mixture of lignocellulosic substrate and synthetic polymer (Figure 5f,g) will form the matrix structure to sustain the fungal material, ensuring the application as thermal insulating material for buildings.

### 3.5. Morphological Analysis of Fungal Biomaterial

**Figure 6:**
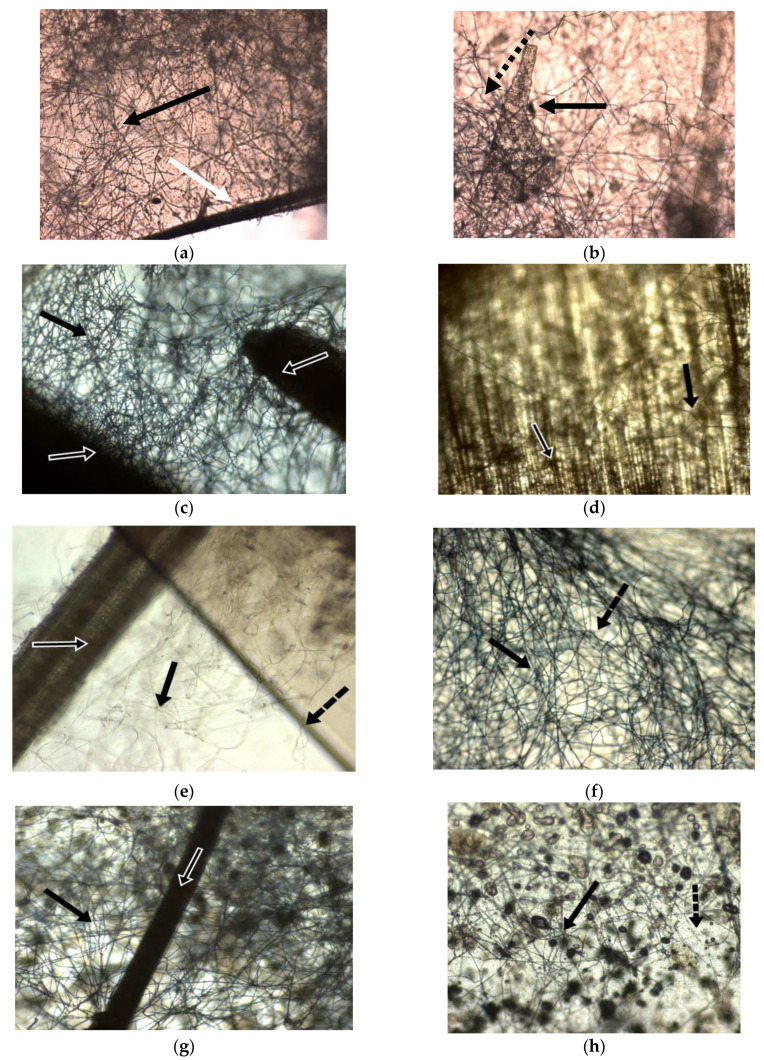

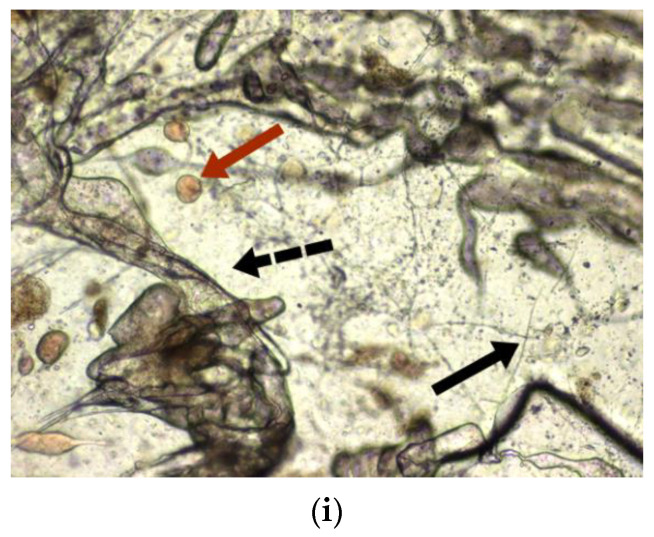
Morphology of *Ganoderma* cultures on lignocellulosic substrate (wheat straws) and polypropylene embedded with bacterial spores. (**a**) Hyphae network of *Ganoderma* (black arrow) and wheat straws (white arrow) (10×); (**b**) Hyphal network of *Ganoderma* (black arrow) and polypropylene (black dotted arrow) (10×); (**c**) Wheat straws (white arrow) and hyphae network of *Ganoderma* (black arrow) (10×); (**d**) Wheat straws (white arrow) covered by hyphae network of *Ganoderma* (black arrow) (10×); I A wheat straw (white arrow), network of fungal filaments of *Ganoderma* (black arrow), propylene piece (black dotted arrow) (10×); (**f**) Polypropylene piece (black dotted arrow) covered by hyphae network of *Ganoderma* (black arrow) (10×); (**g**) A wheat straw (white arrow) and network of fungal filaments of *Ganoderma* (black arrow) (10×); (**h**) Small polypropylene piece (black dotted arrow) covered by fungal filaments of *Ganoderma* (black arrow) (10×); (**i**) Polypropylene (dotted black arrow), agglomerations of *Bacillus* spores. (red arrow) and fungal filaments from *Ganoderma* mycelium (black arrow) (10×).

During incubation, the fungal cultures were visually evaluated for mycelium coverage consistency, as well as for detection of any infection. The mycelium colonization of substrate was macro- and microscopically inspected throughout incubation. The morphology images of fungal cultures are presented in Figure 6.

Light microscopy showed that *Ganoderma lucidum* had dense and abundant culture, with hyphal networks spread on surface and between straws and polymeric fragments (Figure 6a–c). The wheat straws represent a good nutrient substrate for *Ganoderma*, which is able to secret an enzymatic complex specialized in hydrolyzing lignocellulosic materials. In Figure 6i, it can be observed even bacilli from *Bacillus amyloliquefaciens* embedded in polypropylene.

**Figure 7:**
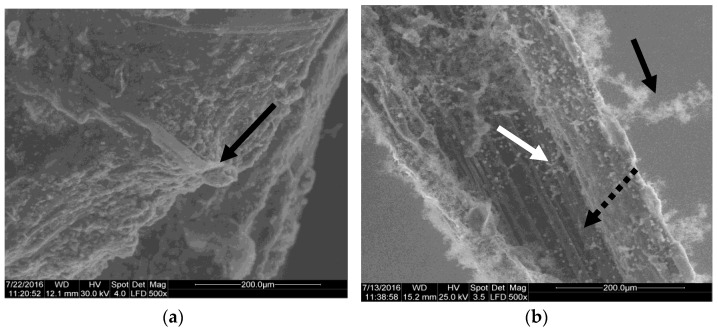

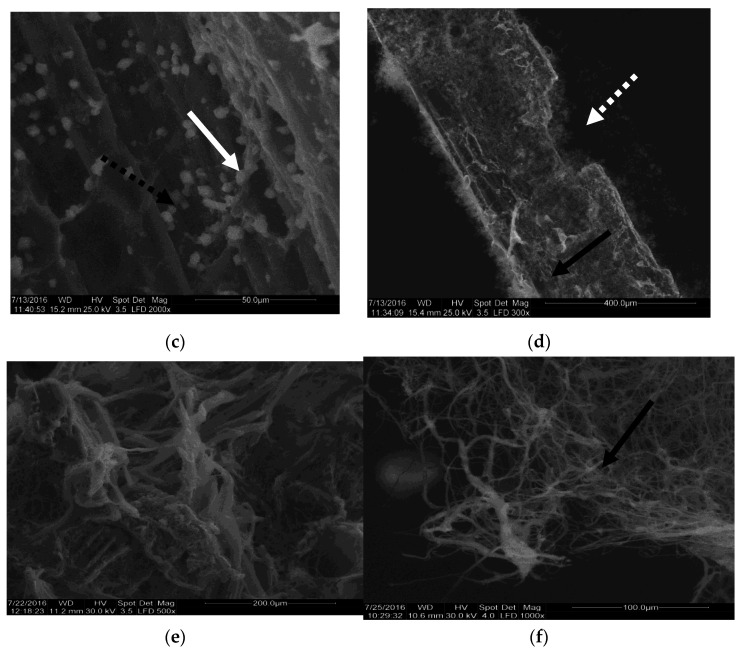
SEM images of *Ganoderma lucidum* cultured on lignocellulosic substrate (wheat straws) and polypropylene embedded with bacterial spores. (**a**) Fungal hyphae (black arrow); (**b**) Mycelium (black arrow); wheat straw structure (dotted black arrow); conidia (white arrow) (500×); (**c**) Wheat straw structure (black dotted arrow); conidia (white arrow) (2000×); (**d**) Fungal mycelium attached to wheat straw (black arrow); degradation of wheat straw (white dotted arrow) (300×); (**e**) Ribbons or tubular hyphae from mycelium growing on substrate (500×); (**f**) Aerial mycelium detached from substrate (black arrow) (1000×).

SEM observations are presented in Figure 7. Fungal hyphae were growing on substrate structure (Figure 7a). A fluffy mycelium was growing attached or aerial detached to substrate structure, namely, wheat straws (Figure 7b). In Figure 7c it can be seen individual or crowded conidia spread on substrate surface. The fungal filaments appeared as ribbons or tubes (Figure 7e). Thin elongated and branched fibers of fungal hyphae with multiple ramifications and sub-ramifications are observed in Figure 7e,f.

SEM and optical investigations applied to biomaterial samples revealed the lack of any contamination of fungal culture.

### 3.6. Chemical Characterization FTIR Spectrum Analysis

**Figure 8:**
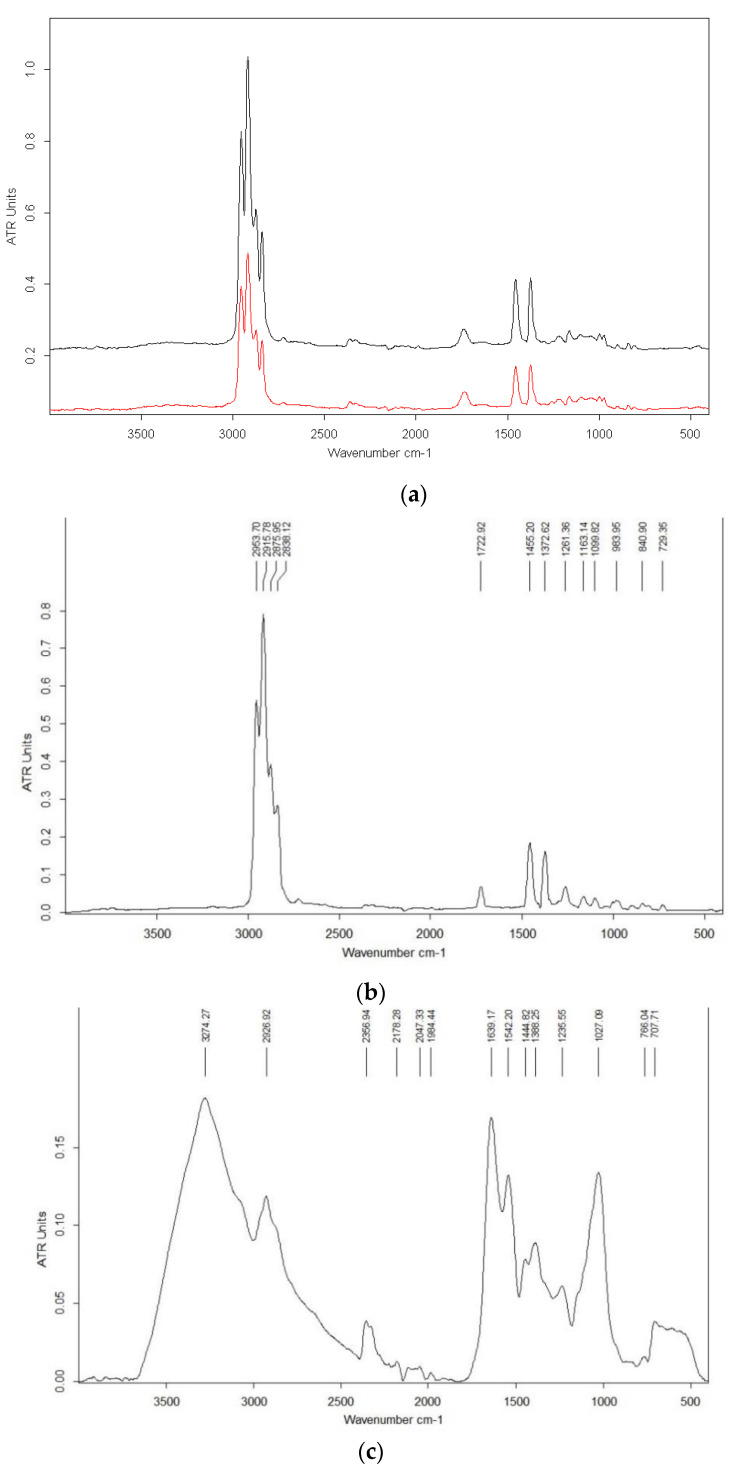

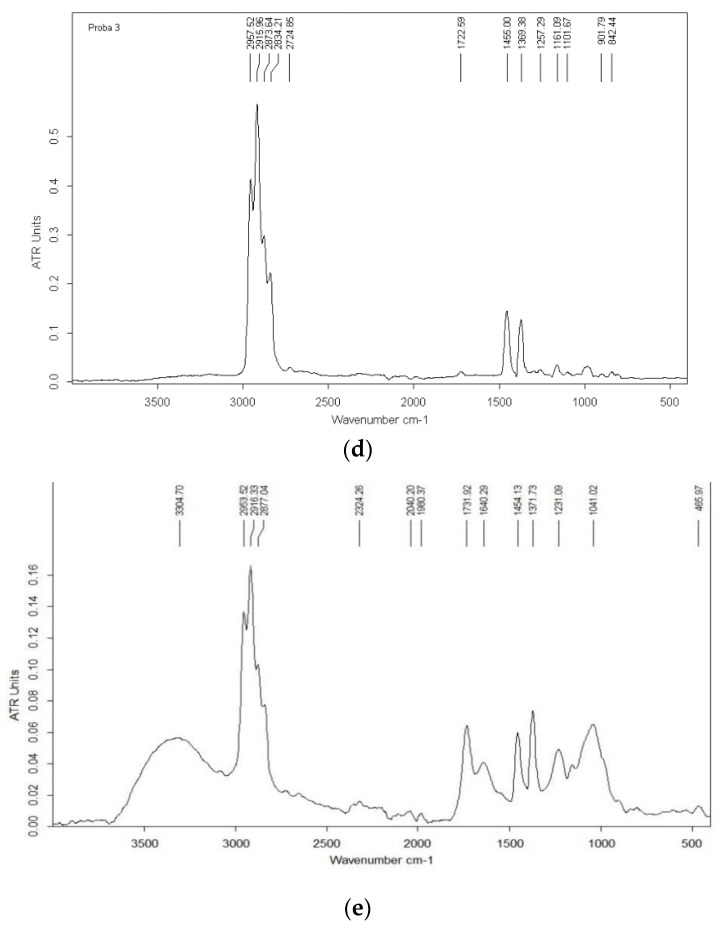
FTIR analysis of biocomposites. (**a**) PP embedded with *Bacillus* spores (red line), virgin PP (black line); (**b**) PP without bacterial spores, before inoculation with *Ganoderma;* (**c**) PP without bacterial spores inoculated with *Ganoderma* (after three weeks of incubation); (**d**) Mixture of wheat straws, PP embedded with bacterial spores, before inoculation with *Ganoderma*; (**e**) Mixture of wheat straws, PP embedded with bacterial spores, inoculated with *Ganoderma* (after three weeks of incubation).

The FTIR spectra were analyzed in order to characterize the chemical composition of the studied biomaterial samples, and to compare the chemical nature of substrates, as can be seen in Figure 8.

Polypropylene presented the specific bands in accordance with literature data [19]: broadband at 2953–2840 cm^−1^ assigned to C-H stretching; 1455 cm^−1^ assigned to C-H_2_ bending; 1372 cm^−1^ assigned to C-H_3_ bending; 1163–1164 cm^−1^ assigned to C-H bending, CH_3_ rocking, C-C stretching; in the range 840–1099 cm^−1^ assigned to CH_3_ rocking, C-C stretching, C-H and C-H_3_ bending (Figure 8a). Also, it can be seen that no significant difference was signaled between PP pure and PP embedded by *Bacillus* spores. Regarding the spectra of mixed samples including wheat straws, PP embedded with bacterial spores and *Ganoderma* mycelium, the broadband between 3000 and 3650 cm^−1^ is due to the presence of OH group in the samples and due to the absorption of water (Figure 8c,e), by comparison with samples without mycelium where these bands are missing (Figure 8b,d). The band around 1640 cm^−1^ is due to C=C stretching or N–H bending (amide I from proteins). The intermediate range at 1370–1390 cm^−1^ is due to O–H (Figure 8b–e). According to several reports, the broadband in the lower region at 1027–1041 cm^−1^ is assigned to carbohydrate and nucleic acid vibrations [9,20,21]. The characteristic functional groups in a fungal mycelium are as follows: 3000–2800 cm^−^^1^ for fatty acids/lipids; 1700–1600 cm^−^^1^ for amide I, 1575–1300 cm^−^^1^ for amide II and amide III (proteins), and 1200–900 cm^−^^1^ for polysaccharides [22].

### 3.7. Thermal Conductivity

**Figure 9:**
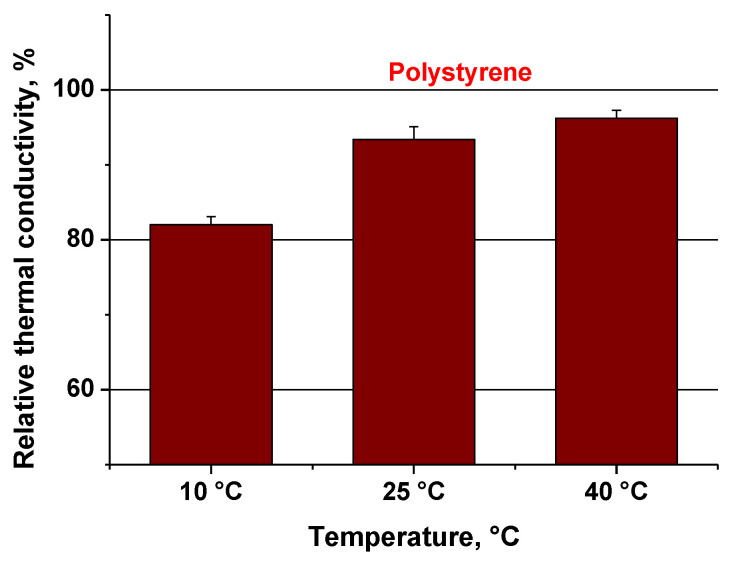
Relative thermal conductivity of bio-polymer composite.

The thermal conductivity of bio-polymer composite based plate was expressed both in W/mK and as a relative value to polystyrene, considered as a reference thermal insulation material (Table 2 and Figure 9).

### 3.8. Mechanical Testing in Compression

Compression strength is an important property in choosing a thermal insulation material. Depending on the application, the thermal insulation materials must have a certain resistance to compression, in order to be able to withstand different loads that can cause damage to the insulation system.

EPS, our reference material, has a cellular structure suitable to withstand compression in applications with different loads.

**Figure 10:**
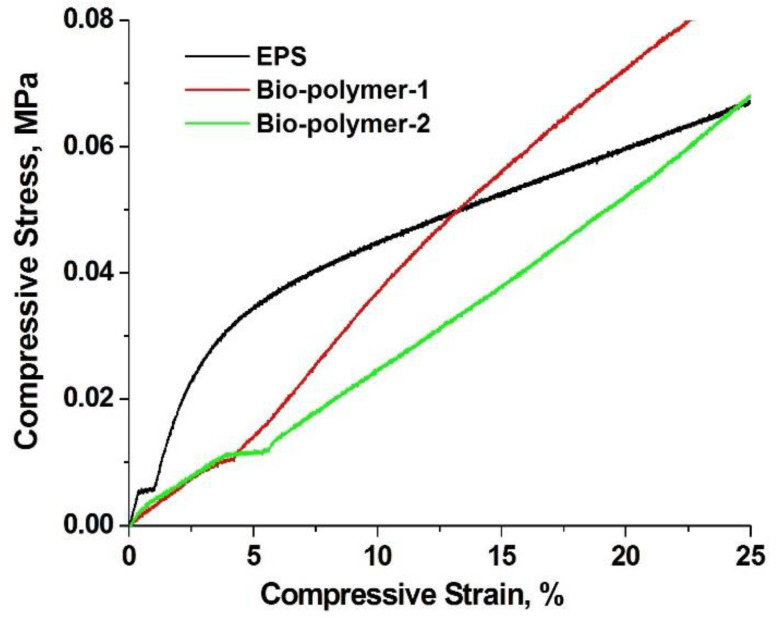
The stress-strain curves of bio-polymer composite in comparison with commercial EPS.

Typically, compressive strength is reported for EPS as 10% deformation. We reported the value of compressive strength for a deformation of 25% because after testing the bio-polymer, we have noticed a completely different behavior from that of EPS and also different from one specimen to another. To better understand the behavior of materials during the compression test, we represented the stress-strain curves (Figure 10). For the bio-polymer we represented the behavior of two from the tested specimens, denoted with Bio-polymer-1 and Bio-polymer-2.

The results for compressive strength, at 25% deformation, are presented in Table 3 and Figure 10, in comparison with those obtained for expanded polystyrene (EPS).

## 4. Discussion

Since there is an increasing interest in developing mycelium based biocomposites, there are many studies focused on the attempt to find new strains able to produce a strong and dense network of fungal filaments. As an example, it was reported a biomaterial including *Trichoderma asperellum* and *Agaricus bisporus*, grown on oat husk and rapeseed cake after oil pressing [23]. A manufacturing system of seven steps was elaborated to produce a sandwich structure of biocomposites made from agriculture waste and mushroom mycelium. In these matrix structure, the mycelium is acting as a “glue” for the whole mixture. The biomaterial demonstrated many advantages over traditional synthetic composites, such as: competitive strength, tensile and impact mechanical properties, reduced energy consumption and biodegradability [24]. The edible mushroom known as “Shiitake”, *Lentinula edodes* was used in the production of composites based on the mixture of polypropylene and sugarcane bagasse [25], or in a mixture of coconut powder with bran [26]. The last approach had the benefit to valorize the residues from coconut industry and to reduce the water and soil contamination. Three fungal species as *Colorius* sp., *Trametes* sp., *Ganoderma* sp. and pruning residues of apple and vine crops were used to prepare a fungal material. The authors described the strong correlation between factors as fungal specie, substrate composition and fermentation conditions and properties of final mycelium-material [27]. A test conducted with *Trametes versicolor* revealed that the mechanical performances of the mycelium based composites depend more on the fiber condition, size, processing, than on the chemical composition. The study evaluated many lignocellulosic substrates (hemp, flax, flax waste, soft wood, straw), processed in different ways (loose, chopped, dust, pre-compressed and tow) [28].

Our proposed fungal biomaterial is based on the fungal mycelium from *Ganoderma lucidum* growing on mixed substrate including lignocellulosic and polypropylene trips embedded with bacterial spores. The interactions between bacteria and fungi are very complex and important for their common life in ecosystems Depending on both microorganisms involved, the interactions could lead to negative or beneficial effects. In some cases, bacterial exopolysaccharide and biofilm formation stimulate the growth of fungal species, the fungi consuming the bacterial cells constituents and assimilating them as nutrients [29,30,31].

The second microorganism *Bacillus amyloliquefaciens* 1014 play a significant role by improving the biocompatibility for thermoplastic materials intended in the formation, reinforcement, increased durability of acoustic or thermal-insulating biomaterials obtained from the mycelium. The choice was based on the fact that *Bacillus amyloliquefaciens* 1014 produces resistant spores protected through various mechanisms that ensure their survival for long period, dormant period, with little or no nutrients. Weather the environment is changing and the amount of nutrients increases, the spores are able to return to life by germination. If the damages of spore’s structure were too strong affecting the core, the germination process is stopped [32]. The mechanism resistance to heat is a multicomponent one, attributed mainly to bacterial spores with low level of water [33]. Similar survival capacity of bacterial spores has been reported [34,35].

The viability of bacterial endospores (spores) is a suitable property for spore incorporation in plastic material for storage of many packed food products. Among the different active oxygen scavengers to incorporated in the polymer matrix, spores from *Bacillus amyloliquefaciens* were found to be able to improve the gas barrier properties of material and also remove residual oxygen. Such a model system was successfully elaborated for poly(ethylene terephthalate) bottles with 4% w/w bacterial spores concentration. After processing temperature, it was shown that material moisture is enough to allow spore rehydration and germination. The incorporation of biological systems seems to be a distinct advantage since the spores can be incorporated direct in polymer matrix compared with the addition of sachets or coatings redundant. It is known that a dormant spore is very small, around 1 μm, while vegetative cell is bigger. So, the spores have not the volume necessary to become a vegetative cell, and meeting the minimal growth conditions they are able to germinate [36].

In this purpose, the bacterial spores from *Bacillus amyloliquefaciens* 1014 were incorporated in the plastic material and tests results showed that the spores are viable even after exposure to heat treatment (Figure 2). Normally, the spores had to migrate to the outside to form hydrophilic biofilms, the outdoor migration and biofilm formation depends on the number of bacterial spores. Since bacteria can only be incorporated within the limit of 5–6%, because otherwise they would change the mechanical characteristics of plastics, a high rate of bio-film formation is therefore necessary for a high survival rate of bacteria incorporated in the materials processed in the extreme conditions (for biological systems) of temperature and pressure. The details regarding the incorporation of bacteria into the plastic material, ensuring a high survival bacterial rate were described in the patent application cited here [3]. The possibility of embedding bacterial spores for better compatibilization of hydrophobic plastic materials offers further possibilities to develop new biocomposite materials.

It is important to underline the lack of antagonism between both selected organisms (Figure 3), allowing the growth of fungus on a mixture of lignocellulosic substrate and polypropylene embedded with bacterial spores.

As a general remark, the bands from FTIR spectra of bio composites belong mainly to polypropylene. The broadband between 3000 and 3650 cm^−1^ is presented only in samples containing lignocellulose, PP embedded with bacterial spores and fungal mycelium, and its presence is due to the OH group present in the samples and due to the absorption of water after fungal cultivation on mixed substrates (Figure 8).

A problem to be solved in future experiments is the relative reduced homogeneity, consequence of mixing chopped wheat straws with polymer strips. The preferred substrate of the fungal strain are lignocellulosics, but it is very difficult to achieve a uniform spread of the polymer strips among the pieces of wheat straw. The fungus grew differently depending on the micro-neighborhood of the inoculation points with *Ganoderma* that is important and can act differently, in a stimulating sense, if it is predominantly lignocellulosic substrate, and relatively restrictive if it is polymer. Also, inside the board, there is an absence of air and an accumulation of heat produced by mycelium growth [28].

The stress-strain curve for EPS (Figure 10) highlights a typical EPS behavior on the compression test [37]. At small compressive strain, below 5%, a rapid increase of compressive stress up to about 35 kPa is observed. Under 2% deformation the increase in stress is linear. Above 35 kPa a continuous increase of stress with deformation was observed (50 kPa for 15% strain and 67 kPa for 25% strain). In the case of bio-polymer-1, a continuous increase of stress with deformation has been observed. Compared to EPS, at the same deformation the stress value is 13–70% lower. Thus, as higher the deformation is, the smaller is the difference in compressive stress (10 kPa compared to 35 kPa for 5% deformation, 38 kPa compared to 50 kPa for 15% deformation and 52 kPa compared to 60 kPa for 20% deformation). Only at 25% deformation the values obtained for compressive stress are similar to those of EPS. The specimen 2 of the bio-polymer sample behaves similarly to sample 1. A continuous, but faster increase in compressive stress with compressive deformation can be observed. At deformations over 12% the stress values exceed the EPS values (56 kPa, 72 kPa and 88 kPa for 15%, 20% and 25% deformation). These results demonstrate an inhomogeneity of the bio-polymer sample. An improvement in the dispersion of the components can lead to a compressive strength similar or even better to that for EPS.

The fungal biomaterial has a better thermal insulation capacity than polystyrene. In comparison with polystyrene, the thermal conductivity of fungal biomaterial is lower by about 4% at 40 °C, 7% at 25 °C and 18% at 10 °C. Even if the values of thermal conductivity were lower than extruded polystyrene, they can be improved by a better homogenization of the mixture and certain changes in the ratio between lignocellulosic and polypropylene pieces. The lack of homogeneity is known to have a significant influence on thermal conductivity of material [29]. In addition, the compressive strength is similar with that of EPS.

We consider that the obtained results are promising for the use of such biomaterials in constructions and it is necessary to continue the investigations by testing other properties (thermal, mechanical) for the field of use.

## 5. Conclusions

The present study can be considered a contribution to the fundamental understanding of fungal biocomposites obtainment. We proposed a composite biomaterial of fungal hyphae networks grown on mixed substrate including lignocellulosics and polypropylene trips embedded with bacterial spores. The growth of *Ganoderma lucidum* led to a light, dense, unshakable and biodegradable structure that allow handling without risk of disintegration. *Ganodema lucidum* and *Bacillus amyloliquefacines* are two common microorganisms, nonpathogenic and easily to be cultured, having multiple practical applications. The material resulted from our work has many advantages, being safe, inert, renewable, natural, green and biodegradable in nature, since it is made of biodegradable materials like wheat straws and fungal mycelium. It should be emphasized that the biomaterial can be obtained in a predefined form, according to desired application.

Despite the fact that polystyrene is a very popular insulating material and widely used, at present, its monomer, styrene, is considered as “priority pollutant” and it is imperative to replace it with safe, non-polluting materials [38]. In this regard, mycelium based materials for insulation sustainable applications could be a viable solution to be applied in construction domain [39].

Accordingly, Girometta et al. (2021) [40] consider the technology for mycelium biomaterials as a model of circular economy because it involves the optimization of resources and energy consumption compared to conventional products, valorization of by-products resulted from production process and biodegradability of products.

Recently, a comprehensive review [41] present the nanocomposites obtained from biorenewable sources, emphasizing aspects as chemistry, structures, advanced applications, and recent developments. As a general conclusion, such materials must have common and clear requirements, namely, easy preparation, low density, low cost, and suitability for modifications, in addition to those specific to the field of use. The authors consider that the major disadvantage in the usage of biorenewable based composites is the formation of microplastics (0.1 μm to 5 mm in size) resulted from rapid biodegradation. These microplastics are similar to those produced from petroleum plastics degradation and the negative effect on environment still remain. The elaboration of composites with higher or total biodegradability degree will be solution of the problem. In future, one of the essential research areas will be the preparation of composites with industrial applications [41].

Taken together the obtained data, the cultivation of edible fungi *Ganoderma lucidum* could represent an ecological alternative for the production of composites for construction domain, when compared commercial products that are currently made of non-renewable resources. In addition, the valorization of agro-industrial by products, the mild conditions and lack of environment contamination during the process, all of which recommend such biomaterials for applications in construction sector. Further investigations are required to improve the homogeneity of mixture in order to obtain the mycelium material with a wide range of potential performances and functions.

## Figures and Tables

**Table 1:** Ratio of components in atomized mixtures.

Variants	Maltodextrin (g)	Bacterial Pellet (g)	Phosphate Buffer (mL)
I	10	10	80
II	10	30	60
III	30	10	60

**Table 2:** Thermal conductivity of bio-polymer composite.

Sample	Thermal Conductivity (W/mK)		
	10 °C	25 °C	40 °C
Bio-polymer composite (vheat straws, PP with bacterial spores and *Ganoderma* mycelium)	0.029 ± 1.04	0.034 ± 1.68	0.035 ± 1.05

**Table 3:** Compressive strength of bio-polymer composite in comparison with commercial EPS.

**Compressive Strength, at 25% Deformation**	**Value, kPa**	
	**Bio-Polymer**	**EPS**
	70 ± 0.01	70 ± 0.01
